# *Plasmodium falciparum *parasites causing cerebral malaria share variant surface antigens, but are they specific?

**DOI:** 10.1186/1475-2875-9-220

**Published:** 2010-07-27

**Authors:** Nabila Kheliouen, Firmine Viwami, Francis Lalya, Nicaise Tuikue-Ndam, Else C Eboumbou Moukoko, Christophe Rogier, Philippe Deloron, Agnès Aubouy

**Affiliations:** 1 Institut de Recherche pour le Développement (IRD) UMR216, Mother and Child faced with tropical infections Unit, Paris, 75006, France; 2 Université Paris Descartes, Faculté de Pharmacie, Paris, 75270, France; 3 UMR216 IRD, Cotonou, Benin; 4 Paediatric Department, Centre National Hospitalo-Universitaire (CNHU), Cotonou, Benin; 5 Unité de recherche en biologie et épidémiologie parasitaires, Institut de Recherche Biomédicale des Armées - Antenne de Marseille & Unité de recherche en maladies infectieuses et tropicales émergentes, UMR6236, Marseille, France; 6 Département des Sciences Biologiques, Faculté de Médecine et des Sciences Pharmaceutiques, Université de Douala, BP2701, Douala, Cameroun

## Abstract

**Background:**

Variant surface antigens (VSA) expressed on the surface of *Plasmodium falciparum*-infected red blood cells constitute a key for parasite sequestration and immune evasion. In distinct malaria pathologies, such as placental malaria, specific antibody response against VSA provides protection. This study investigated the antibody response specifically directed against VSA expressed by parasites isolated from individuals presenting a given type of clinical presentation.

**Methods:**

Plasma and isolates were obtained from four groups of Beninese subjects: healthy adults, patients presenting uncomplicated malaria (UM), cerebral malaria (CM), or pregnancy-associated malaria (PAM). The reactivity of plasma samples from each clinical group was measured by flow cytometry against parasites isolated from individuals from each clinical group.

**Results:**

Antibody responses against VSA_UM _were predominant in CM, UM and HA plasmas. When analysed according to age in all plasma groups, anti-VSA_CM _and -VSA_UM _antibody levels were similar until six years of age. In older groups (6-18 and >19 years of age), VSA_UM _antibody levels were higher than VSA_CM _antibody levels (*P *= .01, *P *= .0008, respectively). Mean MFI values, measured in all plasmas groups except the PAM plasmas, remained low for anti-VSA_PAM _antibodies and did not vary with age. One month after infection the level of anti-VSA antibodies able to recognize heterologous VSA_CM _variants was increased in CM patients. In UM patients, antibody levels directed against heterologous VSA_UM _were similar, both during the infection and one month later.

**Conclusions:**

In conclusion, this study suggests the existence of serologically distinct VSA_CM _and VSA_UM_. CM isolates were shown to share common epitopes. Specific antibody response to VSA_UM _was predominant, suggesting a relative low diversity of VSA_UM _in the study area.

## Background

In areas of intense *Plasmodium falciparum *transmission, protective immunity to malaria is gradually acquired during childhood, leading to decreased susceptibility at adulthood. Immunological protection against parasite blood stages is mainly antibody mediated [1,2]. Among protective antibodies are immunoglobulin G antibodies with specificity for variant surface antigens (VSA) expressed on the surface of *P. falciparum*-infected red blood cells (IRBC) [3]. Their level of expression increases with age, in relation to the endemicity of the area [4]. Clinical disease is thought to be the consequence of infection by parasites expressing VSAs that are not recognized by preexisting VSA-specific antibodies in the infected individual. Each new parasite infection induces a variant-specific antibody response, with specificity for the VSA expressed by the infecting parasite [4-6]. Unfortunately, an immense level of diversity among the *var *genes was shown, although evidence of geographic structuring of variation emerged in isolates causing pregnancy-associated malaria (PAM) [7]. PAM mostly occurs in primigravidae, as they lack antibodies against the particular variant parasite population adhering to the placenta, and expressing PAM-specific VSAs [8,9]. Similarly, VSAs expressed by parasites isolated from children presenting with severe disease (VSA_SM_) were described as more commonly recognized than VSA expressed by parasites isolated from children with uncomplicated malaria (VSA_UM_), suggesting that distinct serological groups are related to the clinical status of the infection [10-12].

VSAs contribute to the sequestration of IRBC in deep organs via the binding to endothelial receptors. This mechanism enables the parasites to avoid splenic clearance [13]. In PAM, IRBC expressing VAR2CSA (the major VSA specifically expressed by PAM parasites) adhere to the placenta [14,15]. Severe malaria leads to a wide range of clinical symptoms categorized in cerebral malaria (CM), severe malarial anaemia, and respiratory distress [16,17]. The pathophysiology of CM includes cytoadherence of IRBC to endothelial cells, and the accumulation of IRBC in brain capillaries was displayed by electron microscopy [17,18]. The implication of cytoadherence in the two other severe malaria types is not so obvious, although the occurrence of mixed clinical types of severe malaria is not rare. Our hypothesis assumes that the clinical status of a malaria infection is related to the specificity of the VSA expressed on the IRBC. Although a limited number of studies have been carried out to study antigenic variation in isolates obtained in CM patients [11,19,20], VSA_CM_, as a subgroup of VSA_SM_, is likely to be relatively conserved due to restricted functional specialization for high binding capacities in brain capillaries. In the current study, the antibody response specifically directed against VSA expressed by parasites was investigated. Parasites were isolated from a given type of clinical presentation, cerebral, uncomplicated, or pregnancy-associated malaria.

## Methods

### Study areas and patients enrolment

Patients were enrolled in Cotonou (6° 21' 35'' North, 2° 26' 23'' East) and Allada (6°39'52'' North, 2°09'35'' East) in southern Benin over one malaria season (April to September 2008). Allada is about 40 km north of Cotonou. In southern Benin, malaria is mesoendemic and transmission is perennial (Damien *et al*., submitted). The transmission level in Cotonou was characterized in 1992 by 33 infecting bites per man per year [21]. Severe malaria mainly occurs during the rainy season (April to mid-July). Cerebral malaria (CM) patients and pregnant women were included in two health facilities in Cotonou, the National Hospital (CNHU, Centre national hospitalo-universitaire) and the main maternity hospital (HOMEL, hôpital mère-enfant de la lagune), respectively. Uncomplicated malaria (UM) patients were enrolled in two schools located at Allada in children more than five years of age attending for school nurseries. Healthy adults (HA) were recruited in the blood donation centre in CNHU. All patients were included after written informed consent from themselves or their guardian. Patients were managed by the medical team of each health facility. An adequate anti-malarial treatment was administered according to the national malaria treatment policy. Ethical clearance was obtained from the Institutional Ethics Committee of the Faculté des Sciences de la Santé at the Université d'Abomey-Calavi in Benin.

### Clinical and biological diagnostic

Three groups of patients presenting distinct clinical malaria status were enrolled: uncomplicated malaria (UM), cerebral malaria (CM) and pregnancy-associated malaria (PAM). Briefly, UM was defined as the combination of fever (tympanic temperature ≥ 37.8°C) or a history of fever in the preceding 24 hours, confirmed presence of *P. falciparum *infection, and absence of any severity sign as defined by the WHO [22]. CM was defined by a Blantyre score at diagnosis ≤ 2 combined with a coma duration of six hours at least, and confirmed presence of *P. falciparum *infection with exclusion of other cause for coma, particularly meningitis. PAM cases were obtained in pregnant women just before delivery. Patients were all screened for malaria infection by rapid diagnostic test for *P. falciparum *(Malaria Quick test, Cypress Diagnostics, Langdorp, Belgique). A questionnaire was administered to the children' guardian, or the pregnant women to collect social data, clinical signs and treatment received before hospital admission.

Two additional groups of subjects were added as plasma controls, healthy male adults (HA) living in Cotonou, and non-immune healthy French volunteers (NI) living in France and having never been exposed to malaria. Peripheral venous blood sample (5 ml) was collected from all study individuals in vacutainer tubes containing EDTA. *Plasmodium falciparum *infections were confirmed by Giemsa-stained thick blood films, and parasitaemia was quantified by counting against 200 leucocytes assuming a mean of 8,000 leucocytes per millimeter of blood. Blood group was determined for each blood sample. Plasmas were separated and frozen from all subjects. Parasite isolates from all malaria-infected patients with O blood group were frozen. Blood count and lumbar puncture for meningitis diagnostic were achieved for patients from the CM group.

### Parasite isolates and *in vitro *maturation

Infected red blood cells obtained from patients with O blood group were snap-frozen in a glycerolyte solution, as described [23]. Twenty-six *P. falciparum *isolates from patients (7 in the CM group, 7 in the PAM group, and 12 in the UM group) were frozen at -150°C. Cryopreserved IE were subsequently thawed and matured *in vitro *for 24-36 hrs [24]. The multiplicity of infection (MOI) was estimated by PCR typing of the polymorphic regions of the genes encoding merozoite surface protein-1 (*msp1*) and merozoite surface protein-2 (*msp2*), as described previously [25]. The MOI was estimated as the largest number of alleles observed between the two loci analysis.

### Plasma samples

A total of 256 plasma samples were collected as follows: 127 samples were collected at the time of malaria diagnosis (D0), 41 from CM patients, 62 from UM patients, and 24 from women with PAM. CM and UM patients were also seen at convalescence (D30), 17 plasma samples were obtained in the CM group, and 62 in the UM group. The high number of lost to follow up in the CM group is mainly explained by the urban localization of the children enrolled, and the difficulty to get them back one month after a severe malaria attack. Two sets of control plasmas were obtained from 44 healthy adults (HA) living in Cotonou, and from six French volunteers never exposed to malaria (NI).

### Typing of VSA expression by flow cytometry

The fluorescence-activated cell sorter (FACS) analysis was used for measurement of specific anti-VSA antibody, as described before [26]. Briefly, thawed parasites were matured to late stages (late trophozoites and schizontes) 24-36 hrs at 37°C in a candle jar, and enriched by exposure to a strong magnetic field (MACS, Miltenyi Biotec, Paris, France). Aliquots of 2 × 10^5 ^IRBC in PBS-FCS 2% were labeled with ethidium bromide (0.002 mg/ml) to allow flow cytometric exclusion of remaining uninfected erythrocytes, and with 100 μl of goat anti-human IgG - FITC (IM1627, Beckman Coulter, Nyon, Switzerland). Labeled IRBC were sequentially exposed to 5 μl of plasma. Samples were analysed from a minimum acquisition of 5,000 IRBC using a Facscalibur cytometer (Becton Dickinson). The level of IgG recognizing VSAs was expressed as mean fluorescence intensity (MFI) in the channel for ethidium bromide-labeled IRBC. Nonspecific labeling was evaluated by analysis of uninfected (ethidium bromide-negative) erythrocytes. All plasma samples tested against a particular parasite isolate were processed and analysed in a single assay. NI plasmas samples were tested against all isolates. However, due to limitations in amounts of IRBC available, not all plasma samples were tested against all parasite isolates (Table 1). To be able to compare VSA IgG levels between isolates and plasma samples, the mean plus two standard deviations of log MFI values obtained with the six NI samples was subtracted to all log MFI values obtained with each isolate. The threshold for positivity was defined as two standard deviations above the mean MFI from the six NI samples.

**Table 1:** Number of isolate-plasma combinations analysed by FACS.

	Clinical type of isolate donors		
Clinical type of plasma donors	UM (n = 12)	CM (n = 7)	PAM (n = 7)
UM D0 (n = 62)	139	95	104
UM D30 (n = 62)	137	117	103
CM D0 (n = 41)	194	104	146
CM D30 (n = 17)	82	58	63
PAM (n = 24)	194	131	129
HA (n = 44)	183	120	134

UM, uncomplicated malaria; CM, cerebral malaria; PAM, pregnancy-associated malaria;

HA, healthy adults; D0, Day of malaria diagnosis; D30, one month after diagnosis.

### Statistical analysis

Data were analysed by using the Statview software programme. For analyses performed with data obtained the day of diagnosis, data obtained with homologous plasma/isolate pairs were excluded. The levels of antibody responders were compared using the χ^2 ^test. VSA Ab recognition was evaluated by analysis of covariance (ANCOVA), two-factor ANOVA, t-test and Wilcoxon rank test for paired data. Clonality and *msp *family prevalence were compared between clinical groups by Kruskall-Wallis and Mann-Whitney U-tests. Spearman correlation test was used to estimate MOI and parasite density correlation. Values of *P *< 0.05 were considered statistically significant.

## Results

### Clinical and biological categorization of patients

A total of 127 malaria-patients were enrolled. Three groups of patients were identified as CM (n = 41), UM (n = 62), and PAM (n = 24) patients, as well as one group of 34 malaria-exposed healthy male adults (HA). CM patients were between six months and seven years of age with a mean (± SD) of 3.3 (± 1.7) years. UM patients were aged 9.3 (± 2.1) years, PAM women were aged 24.9 (± 6.4) years, and HA were aged 35.1 (± 8.9) years. In PAM women, gravidity varied between 1 and 5 (mean 1.9 ± 1.2), 13 were primigravidae (PG) and 11 were multigravidae (MG). Parasitaemia was highly diverse between and within groups: CM, median (interquartile range) of 32,653 (2,082 - 64,846) per μl of blood; UM, 3,555 (516 - 16,285)/μl; PAM, 17,391 (400 - 88,379)/μl. Mean tympanic temperature (± SD) at inclusion was of 38.8 (± 1.2) for the CM patients, 37.2 (± .7) in UM patients and 38.0 (± .9) in pregnant women.

The CM patients were all comatose, admitted at hospital for at least two hours and presented a mean Blantyre score (± SD) of 1.8 ± 0.46 (min. 0, max. 2). The coma lasted 2.5 days on average (min. 1, max. 9). Meningitis was excluded for all children. Anaemia characterized the CM group with a mean haemoglobin level of 6.6 g/L (± 1.5) and a mean hematocrit of 20.8 (± 4.5). Erythrocytes count was low with 2.7 (± .6) T/L although the mean globular volume and the mean haemoglobin corpuscular content were normal. Respiratory distress occurred in 4/39 (10.3%) children of the CM group. Among the children suffering of CM, six died and five came out of hospital without medical consent. At that time among these five children, two presented a deep coma, two a normal consciousness and the data was missing for one.

### The levels of clonality were similar whatever the clinical origin of the isolates

Means (CI95%) for MOI were respectively 1.49 (0.6 - 3.6), 1.61 (0.6 - 3.1) and 1.83 (0.5 - 4.7) for CM, UM and PAM isolates (*P *= .73 by Kruskall-Wallis test). However, MOI increased with parasitaemia (*P *= .001, rho = .307). The prevalence rates of the allelic families for all isolates were 65%, 27% and 40% for the K1, MAD20 and RO33 families of the *msp1 *locus, respectively, and 94% and 7% for the 3D7 and FC27 families of the *msp2 *locus, respectively. Interestingly, PAM isolates presented a higher frequency for MAD20 and FC27 alleles (48% and 23%, *P *= .04 and *P *= .009, respectively), and a lower frequency for 3D7 alleles (76.5%, *P *= .003) than other clinical groups of isolates. RO33 alleles were more frequent in UM isolates than in other groups (*P *= .05).

### The level of anti-VSA antibody reactivity varies with the clinical origin of the isolates tested

**Figure 1:**
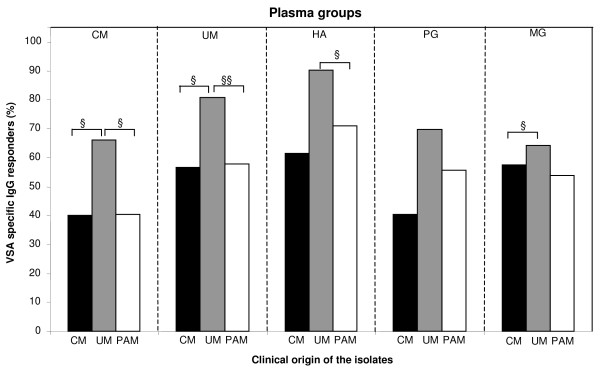
**Levels of specific antibody responders to VSA_CM_, VSA_UM _and VSA_PAM_**. Antibody response was measured in different plasma groups against heterologous *P. falciparum *isolates identified by their clinical origin. CM: cerebral malaria, UM: uncomplicated malaria, HA: healthy adults, PG: primigravidae, MG: multigravidae. Groups were compared by χ^2 ^test (** P < .005, * P < .05, ns. non significant).

The levels of anti-VSA antibody responders were compared against each type of isolates in plasmas of the different clinical groups of patients at the day of diagnosis. Among CM, UM and HA plasma groups, these levels were different between the three origins of isolates (see Figure 1). CM and UM plasmas presented similar patterns of anti-VSA responses. Both plasma types were greater antibody responders against VSA_UM _than against both VSA_CM _and VSA_PAM _(*P *< .05 for all, *P *= .0006 for VSA_UM _versus VSA_PAM _in UM plasmas). In HA plasmas, antibody responses against VSA_UM _were higher than VSA_PAM_. The primigravidae (PG) plasmas contained similar antibody responses to the different VSA types. In multigravidae (MG) plasmas, responses were higher to VSA_UM _than to VSA_CM_. The levels of anti-VSA_PAM _antibody responders in MG and PG plasmas were similar. The quantitative results are also available (see Additional file 1), showing all the specific parasite/plasma combinations tested and including the non immune plasma used as controls in the qualitative analysis.

### Acquisition of anti-VSA antibodies with age varies with the clinical origin of the isolates

**Figure 2:**
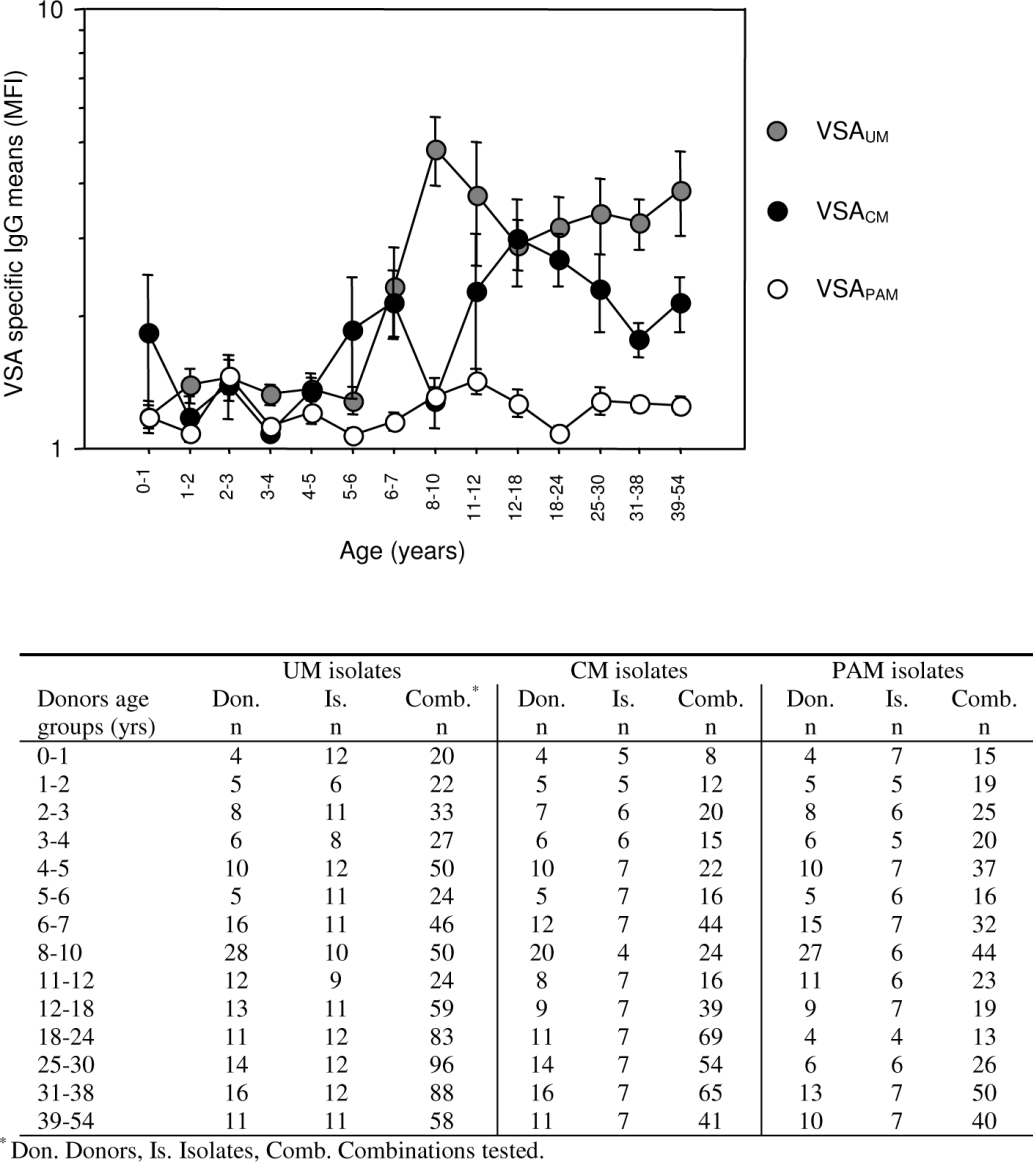
**Mean levels of anti-VSA IgG in Beninese plasma with age, according to the type of isolate tested**. The adjacent table specifies the number of different plasma donors, isolates and the total combinations tested for each age group by type of isolate. For UM and CM isolates, the plasma included come from CM, UM, PAM patients and healthy adults. For PAM isolates, the plasma analysed do not include plasmas from PAM women. Error bars indicate standard errors. Homologous samples are not included in the analysis.

The level of anti-VSA antibodies was analysed at different ages in all plasma groups, except for anti-VSA_PAM _Abs that were measured only in UM, CM, and HA plasmas (Figure 2). Anti-VSA_UM _antibodies levels remained at baseline level until 6 years of age, then increased until 12. From that time, a plateau was reached and maintained until 54 with a mean MFI value around 3.5. Overall, MFI levels for anti-VSA_UM _Abs increased with age (ANCOVA, *P *< .0001). Anti-VSA_CM _antibodies gradually increased until 18 years then decreased, although values did not vary statistically. Mean MFI values remained low for anti-VSA_PAM _Abs (max. 1.5) and did not vary with age. VSA_UM _and VSA_CM _antibody levels were then compared according to three age groups: until 6 years old, between 6 and 18, older than 18. Until 6 years old, levels were similar (t-test). In both other age groups, VSA_UM _antibody levels were higher than VSA_CM _antibody levels (*P *= .01, *P *= .0008, respectively).

### Evolution of anti-VSA_UM_, and anti-VSA_CM _IgG during the first month after infection: the CM isolates share some VSAs

**Figure 3:**
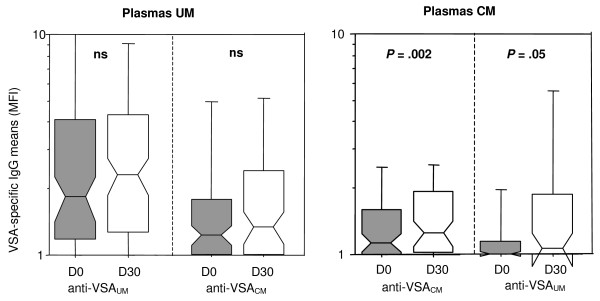
**Acquisition of VSA-specific IgG during the month following *P. falciparum *infection in plasmas samples from UM (n = 121) and CM (n = 56) patients**. UM (A, B) or CM (C, D) plasma samples were tested against *P. falciparum *isolates from UM patients (A, D) or from CM patients (B, C). Centerlines indicate medians, boxes indicate the 25^th ^and 75^th ^percentiles of data points, bars indicate the 10^th ^and 90^th ^percentiles and circles are outliers. Differences are derived from the Wilcoxon rank test for paired comparisons. ns means non significant.

First, MFI values obtained in plasma samples the day of diagnosis and one month later were compared. Plasmas from the same patients were tested against the isolate infecting these patients. For UM plasmas and UM isolates, 10 pairs of results were obtained with both D0 and D30 plasma samples for the homologous isolate. As expected, the levels of anti-VSA_UM _antibodies had increased at D30 (Wilcoxon rank test, *P *= .01). For CM plasmas and CM isolates, only two pairs with both MFI at D0 and D30 for the homologous isolate were available and both showed increased D30 values (see Table 2). Afterwards, the paired MFI values obtained at both Day 0 and at Day 30 in UM or CM plasmas against all UM or CM isolates, were compared, excluding homologous isolate-plasma pairs tested before (n = 125 UM, n = 55 CM) (Figure 3). Interestingly, UM plasmas exhibited similar levels of anti-VSA_UM _antibodies at D0 and one month later (panel A), whereas CM plasmas exhibited higher anti-VSA_CM _antibodies levels at D30 (panel C, *P *= .002). The same analysis was performed after matching ages in UM and CM groups, i.e. with children aged between five and seven years of age. Results were similar as before, anti-VSA antibodies significantly increased at D30 in the CM plasmas (*P *= .033), but not in the UM plasmas (*P *= .206). The same type of analysis was performed to test the potential cross-acquisition of anti-VSA_CM _by UM patients after an UM infection, as well as anti-VSA_UM _by CM patients after a CM infection. No difference was obtained between the anti-VSA_CM _antibody levels displayed at D0 and D30 by UM plasmas (panel B). Conversely, anti-VSA_UM _antibodies were higher in CM plasmas at D30 than at D0 (panel D, *P *= .05). These results show that infection with an isolate of the CM group induced the acquisition of antibodies directed against other CM isolates, but also against UM isolates. No such phenomenon was observed following infection with an isolate of the UM group.

**Table 2:** Plasma recognition of homologous infecting isolate at D0 and D30 for all cases tested at both days.

Plasma type	D0 MFI	D30 MFI
UM*	1.0	2.17
UM	2.26	69.16
UM	1.01	1.71
UM	1.12	6.49
UM	1.44	2.33
UM	2.22	11.44
UM	1.14	1.12
UM	12.39	100.77
UM	1.44	2.83
UM	1.0	1.0

CM*	1.0	1.53
CM	4.76	5.07

Values are mean fluorescence intensities (MFI) measured by flow cytometry.

*UM: uncomplicated malaria, CM: cerebral malaria

## Discussion

This study describes the levels of anti-VSA antibodies displayed by plasmas from different clinical malaria status, uncomplicated, cerebral, or pregnancy-associated malaria, and from healthy adults. The study took place in Benin where malaria transmission level is mesoendemic. Falciparum malaria protective immunity is acquired slowly, according to infections by new parasite variants, until the repertoire of all parasite variants from the area is recognized. Protective antibodies are partly directed against parasite variant surface antigens (VSAs), known to contribute to the cytoadherence of infected erythrocytes to vascular endothelium. In CM and PAM, cerebral capillaries and placenta respectively both concentrate preferentially the IRBC sequestration. In PAM, the existence of a conserved adhesion phenotype in selected parasite variants was proven [14,15]. However, the existence of a specific type of variant surface antigens for cerebral malaria was not shown.

This study includes patient groups categorized by their malaria disease, uncomplicated, cerebral or associated to pregnancy. As the transmission level of the study area is mesoendemic and perennial, these patient classes were also different in age, particularly the CM and younger group. Consequently, anti-VSA antibody responses measured in the CM group were lower compared to the other plasma groups, due to their lesser exposition to malaria. Previous studies showed a more frequent recognition of VSA_SM _than VSA_UM_, and proposed the existence of rare and frequent *falciparum *variants [10,12]. In the present study, Figures 1 and 2 show inverse data. VSA_UM _antibody responses were higher than VSA_CM _antibody responses, although acquisition of both anti-VSA antibody types occurred at about 6 years old, as shown in Figure 2. After the age of 6, levels of anti-VSA_UM _antibodies increased and reached higher levels than anti-VSA_CM_. A high anti-VSA antibody response may reflect three options: i) VSA epitopes are conserved, ii) VSA epitopes are highly prevalent in the area, or iii) VSA epitopes are highly antigenic. The previously cited studies took place in Kenya and Ghana, where transmission intensity is much higher than in southern Benin. These groups proposed that *P. falciparum *favours VSA_SM _variants presenting optimal binding ability. However, whether high virulence is advantageous for parasite population survival is questionable. Indeed, the foundations of parasitism involve the search for equilibrium between parasite and host, i.e. the survival of the host to ensure long-term parasite proliferation. In our understanding, the transmission level of the area allows a certain level for parasite clonal diversity. The level of anti-*P. falciparum *specific immunity certainly constitutes another factor influencing the parasite clonal diversity, as already suggested [27]. In the study area, the prevalence of VSA_UM _antibody response compared to VSA_CM _may be explained by the balance between a relative conservation of VSA_UM_, or relative low clonal diversity, and the level of immunity of the patient groups tested. Epidemiological, entomological and socio-cultural factors that are area-specific are also likely to interfere with clonal composition of *P. falciparum *parasite population.

Importantly, VSA_SM _and VSA_PAM _are thought to be more conserved than VSA_UM_, raising the hope for specific therapies targeting severe types of malaria. This hypothesis can be argued as one month after infection the level of anti-VSA antibodies able to recognize heterologous VSA_CM _variants had increased in CM patients (Figure 3). Although the increase may be due to the disruption of immune complexes, this result shows that VSA_CM _share common epitopes. CM patients in the study were younger than UM patients, and had been probably less exposed to malaria. Consequently, if the infecting isolate was encountered for the first time by CM patients, the VSA_CM _antibody levels logically increased at D30, assuming that CM isolates share similar VSA epitopes. In the UM group, the level of anti-VSA_UM _did not increase one month after the initial infection. These data show that the UM patients have already been exposed to the different VSA_UM _and VSA_CM _tested in the experiment; it does not demonstrate that UM isolates do not share epitopes. Interestingly, the plasmas tested one month after CM infection also contained antibodies capable to recognize VSA_UM _epitopes. Such result was already described with plasmas sampled 28 days after a SM infection [20]. This means that UM and CM isolates share some VSA. The data may also be interpreted by saying that finally VSA_CM _and VSA_UM _do not differ. As the cellular immune response is known to have critical consequences during CM [28], the local response of the host could constitute the major element that decides whether CM will develop or not in a non- or semi-immune host. However, Figure 2 shows that the antibody levels acquired with age differed according to the VSA type tested. These data argue for the existence of serologically distinct VSA_CM _and VSA_UM_. Ultimately, one hypothesis derives from these findings. The prevalence of isolates presenting VCA_CM _may be low in the study area, although they share common epitopes. On the contrary, the prevalence of VSA_UM _isolates may be much higher, and VSA_UM _may not be highly diverse, resulting in a high antibody response specific to anti-VSA_UM _after the age of six.

As expected, CM, UM and HA plasmas weakly recognized VSA_PAM _isolates as shown in Figure 2. This is explained by the rapid elimination of VSA_PAM _variants by the spleen after delivery, once placenta is not present anymore [29]. However, HA and UM plasmas were high antibody responders for VSA_PAM _isolates (Figure 1). These results suggest two hypothesis: i) PAM isolates share common epitopes with non-PAM isolates; ii) their presence in infecting isolates (before a rapid elimination) allows a basal recognition in cytyometry experiments. Additionally, the levels of antibody responses to VSA_PAM _detected in multigravidae plasmas were similar to those measured in primigravidae plasmas. Like the low anti-VSA_CM _antibody response, this result is surprising and suggests again a low prevalence for VSA_PAM _isolates in the area.

## Conclusion

In conclusion, this study suggests the existence of serologically distinct VSA_CM _and VSA_UM_. CM isolates were shown to share common epitopes. Specific antibody response to VSA_UM _was predominant, suggesting a relative low diversity of VSA_UM _in the study area.

## Competing interests

The authors declare that they have no competing interests.

## Authors' contributions

AA, PD and NTN conceived the study and experiments. NK, FV and AA carried out the experiments, except for *msp1 *and *msp2 *study, which was carried out by CR and ECEM. FL organized and supervised the children enrolment and treatment in hospital. AA, PD and NK wrote the manuscript. All authors helped to finalize the manuscript, read and approved the final draft.

## Supplementary Material

Additional file 1 **Aubouy-figureSUPPL-MalariaJ-revised version.** Supplementary figure. Detailed relative levels of VSA specific IgG to heterologous *P. falciparum *isolates according to the clinical origin of the *P. falciparum *isolates, and to the plasma group. Each dot represents a specific plasma/parasite combination. Figures show all combinations tested with CM isolates (A1, A2), UM isolates (B1, B2, B3), and PAM isolates (C1, C2). UM: uncomplicated malaria, CM: cerebral malaria, PAM: pregnancy-associated malaria, PG: primigravidae, MG: multigravidae. Click here for file
