# Supplementary figures and images for: Plasmodium falciparum parasites causing cerebral malaria share variant surface antigens, but are they specific?
Source: Malar J. 2010 Jul 27;9:220. doi: 10.1186/1475-2875-9-220 (PMC2921079; doi:10.1186/1475-2875-9-220)

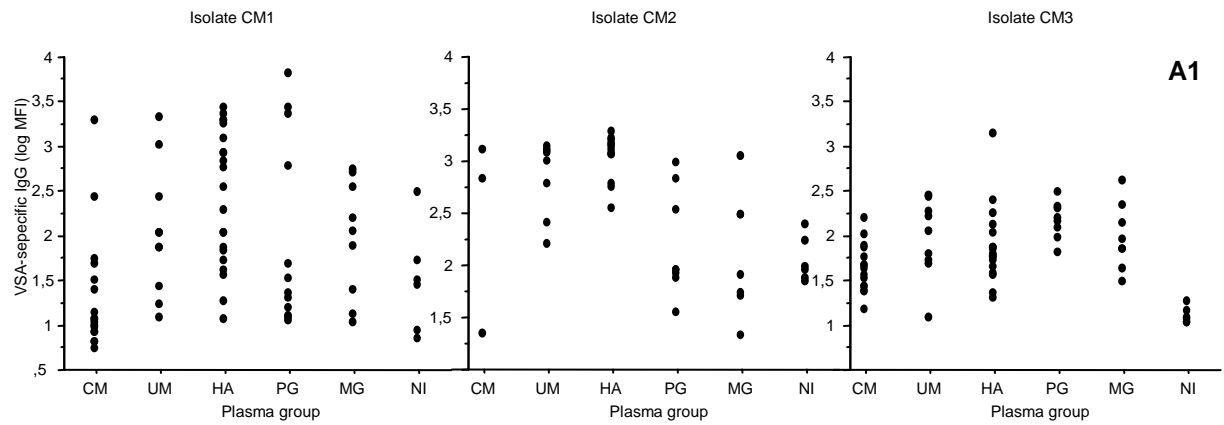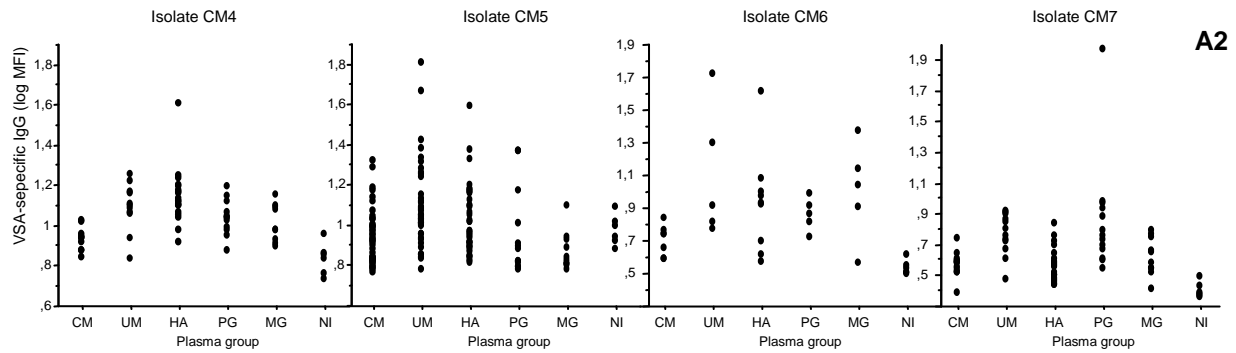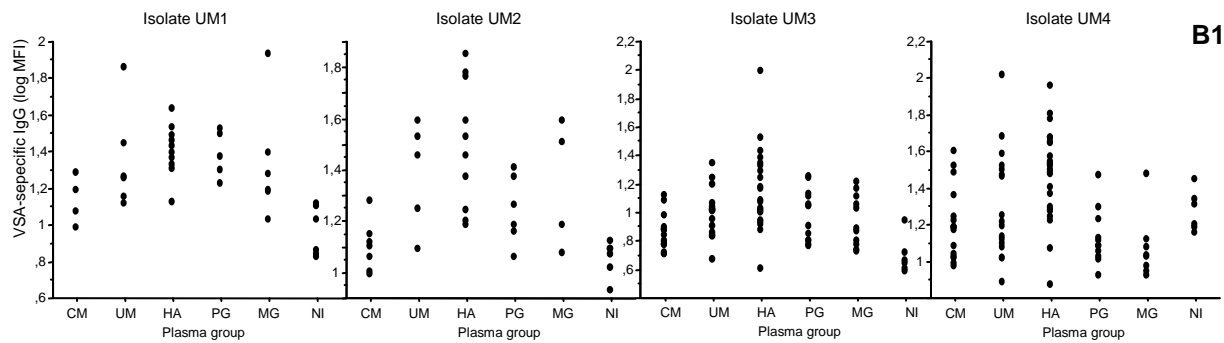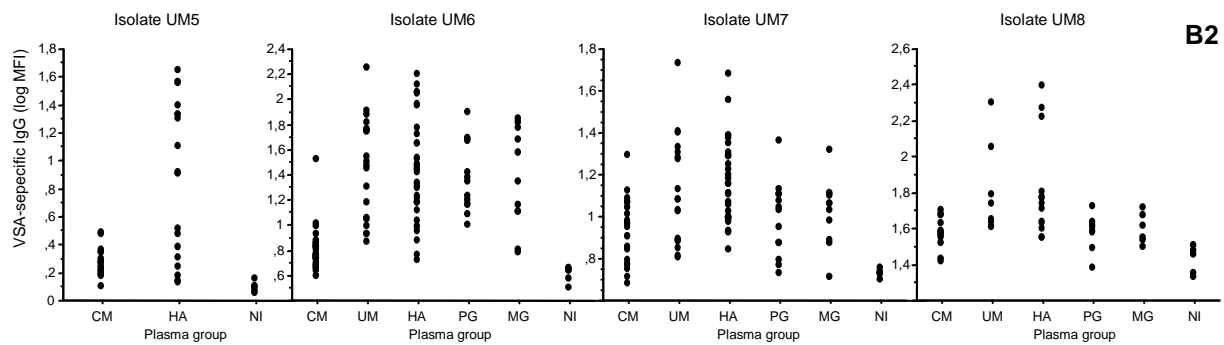

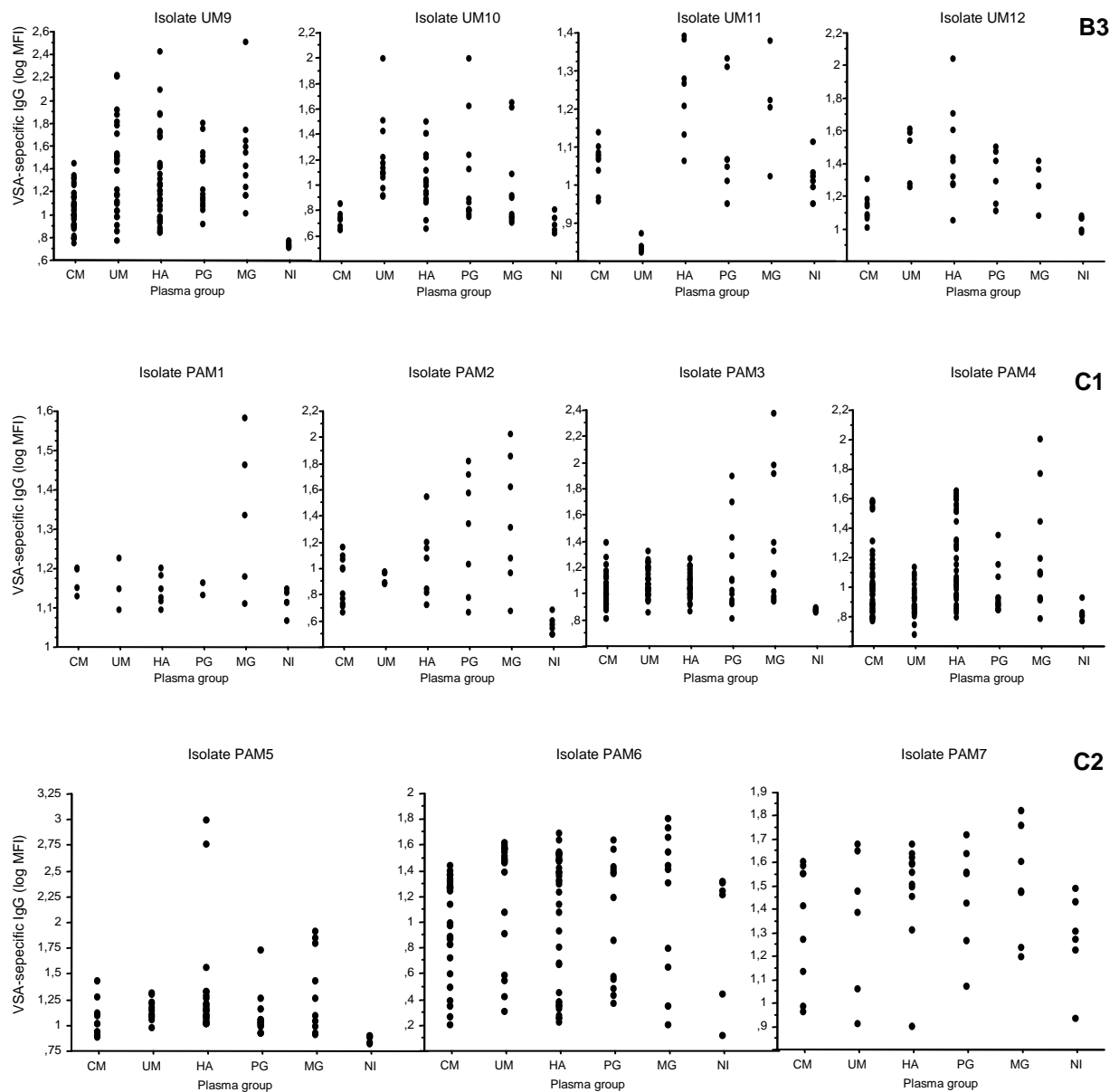

**Supplementary figure.**

Supplement: Additional file 1 — Aubouy-figureSUPPL-MalariaJ-revised version. Supplementary figure. Detailed relative levels of VSA specific IgG to heterologous P. falciparum isolates according to the clinical origin of the P. falciparum isolates, and to the plasma group. Each dot represents a specific plasma/parasite combination. Figures show all combinations tested with CM isolates (A1, A2), UM isolates (B1, B2, B3), and PAM isolates (C1, C2). UM: uncomplicated malaria, CM: cerebral malaria, PAM: pregnancy-associated malaria, PG: primigravidae, MG: multigravidae. [file 1475-2875-9-220-S1.PDF]
